# Successful joint preservation of distal radius osteosarcoma by *en bloc* tumor excision and reconstruction using a tumor bearing frozen autograft: a case report

**DOI:** 10.1186/s12893-018-0346-y

**Published:** 2018-03-01

**Authors:** Takashi Higuchi, Norio Yamamoto, Katsuhiro Hayashi, Akihiko Takeuchi, Kensaku Abe, Yuta Taniguchi, Yoshihiro Araki, Kaoru Tada, Hiroyuki Tsuchiya

**Affiliations:** Department of Orthopaedic Surgery, Graduate School of Medical Science, Kanazawa University, 13-1 Takara-machi, Kanazawa, 920-8641 Japan

**Keywords:** Osteosarcoma, Distal radius, Joint preservation, Liquid nitrogen, Recycled autograft

## Abstract

**Background:**

The wrist joint is an extremely rare site for osteosarcoma. Joint structure preservation to maintain good limb function is well described in case of knee osteosarcoma, whereas it is not described in case of wrist joint osteosarcoma. In this report, we present the first case of joint preservation surgery to treat distal radius osteosarcoma using a tumor bearing autograft treated with liquid nitrogen.

**Case presentation:**

A 46-year-old male presented with swelling and pain in the right wrist and was diagnosed with conventional osteosarcoma of the distal radius. The patient responded well to neoadjuvant chemotherapy and the tumor shrank remarkably. Wide tumor excision to preserve the radiocarpal joint and reconstruction with a tumor bearing frozen autograft were performed. Partial bone union was detected 3 months postoperatively and complete bone union was detected 9 months postoperatively. Following the surgery, there was immediate commencement of the range of motion (ROM) training in both the wrist and fingers. At the final postoperative follow-up of 41 months, the patient had normal ROM in the wrist, fingers, and forearms, with a score of 100% in the Musculoskeletal Tumor Society (MSTS) score and was disease free.

**Conclusion:**

We present the first case in which *en bloc* tumor excision with joint preservation of the wrist and reconstruction using a tumor bearing frozen autograft were performed. The surgery yielded excellent hand, wrist, and forearm function at the final follow-up.

## Background

Osteosarcomas are the most common of primary malignant bone tumors. The most common site of the disease is the metaphyseal bone around the knee [1]. On the other hand, osteosarcoma of the wrist is extremely rare; it has been reported that only < 1% of osteosarcomas arise in the distal radius [1]. Recent advances in multimodality therapy have resulted in limb-sparing surgery being the standard for high-grade osteosarcomas. Furthermore, for select patients, joint-sparing surgery is possible, allowing preservation of the joint structure in an effort to maintain normal limb function [2]. Several case series have reported the usefulness of sparing the knee joint in osteosarcoma in maintaining the function of the knee [3–5], whereas reports regarding wrist joint sparing surgery in osteosarcoma are few. This report presents a successful case of a distal radius osteosarcoma treated by *en bloc* resection and reconstructed with a tumor bearing autograft treated by liquid nitrogen. This surgery resulted in the preservation of the radiocarpal joint, and yielded normal function after the surgery. So, far, this report is the first to describe a wrist joint sparing surgery in distal radius osteosarcoma using a recycled bone graft technique.

## Case presentation

**Fig. 1:**
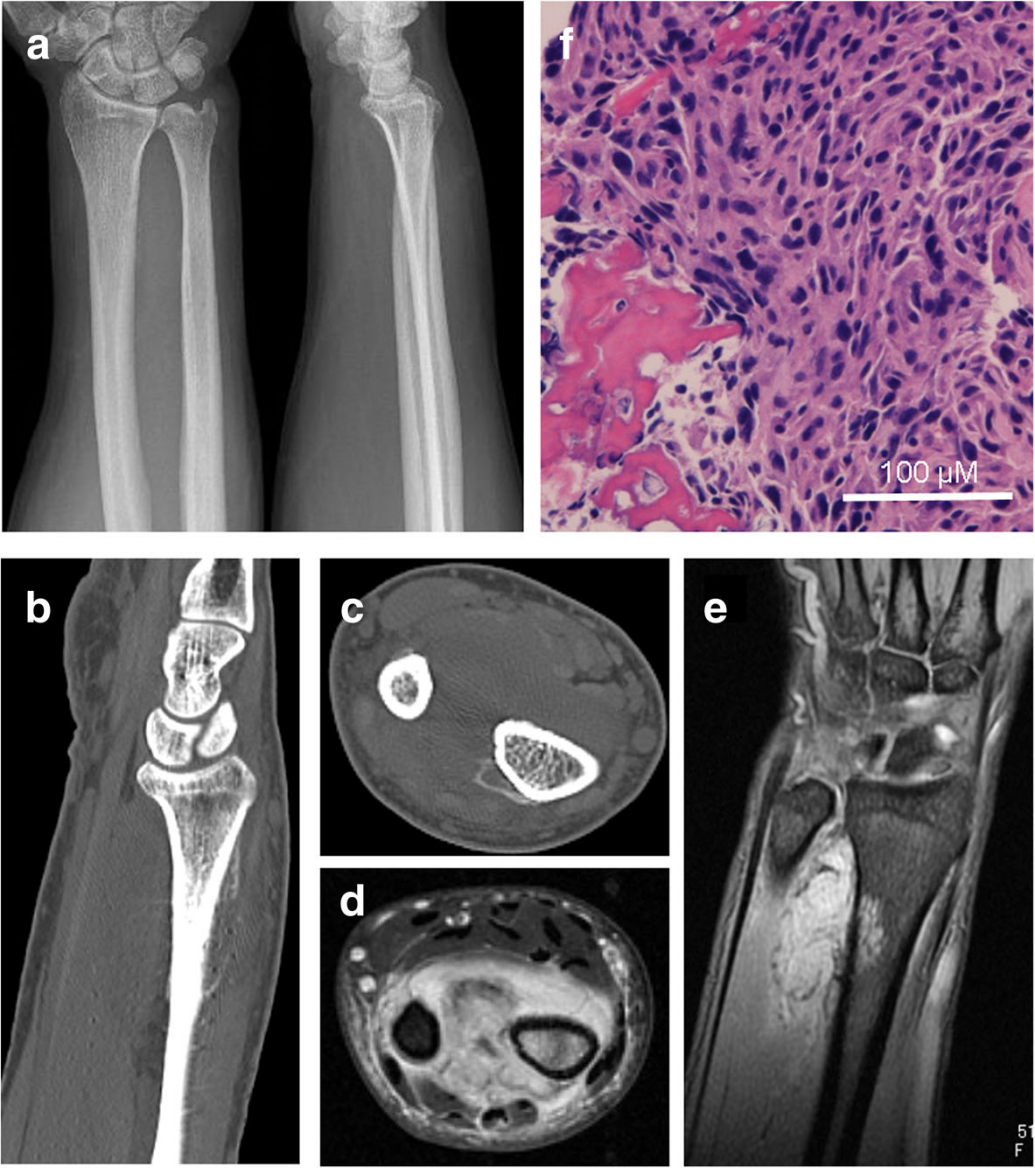
Before neoadjuvant chemotherapy. **a** Plain radiography. Mild cortical irregularity was detected at the ulnar side of the radius. **b**, **c** CT: (**b**) sagittal and (**c**) axial images (bone condition). A periosteal reaction was detected at the dorsal radius. **d**, **e** Gadolinium contrast MRI: (**d**) axial and (**e**) coronal images. A huge extraskeletal tumor surrounding the radius was strongly enhanced. **f** Hematoxylin-Eosin staining of the specimen from open biopsy. Highly dense tumor cells with strong nuclear atypia or atypical mitosis, and tumoral osteoid were detected. No cartilage formation was detected

**Fig. 2:**
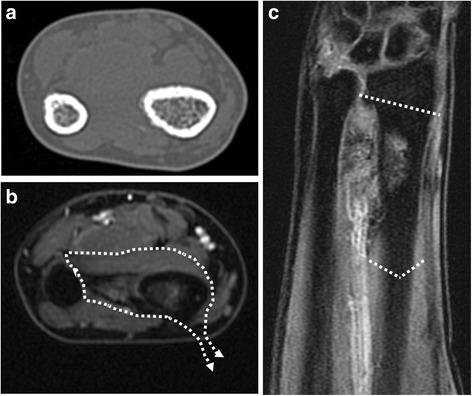
After neoadjuvant chemotherapy. **a** CT axial image (bone condition). Periosteal reaction was detected prior to chemotherapy. **b**, **c** Gadolinium contrast MRI; (**b**) axial and (**c**) coronal images. The soft tissue extension of the tumor shrunk remarkably and a small enhanced lesion was detected in the radius and intraosseous membrane. The dotted arrow indicates the resection range including the radius, intraosseous membrane, and pronator quadratus muscle. The dotted line indicates the osteotomy line of the radius

**Fig. 3:**
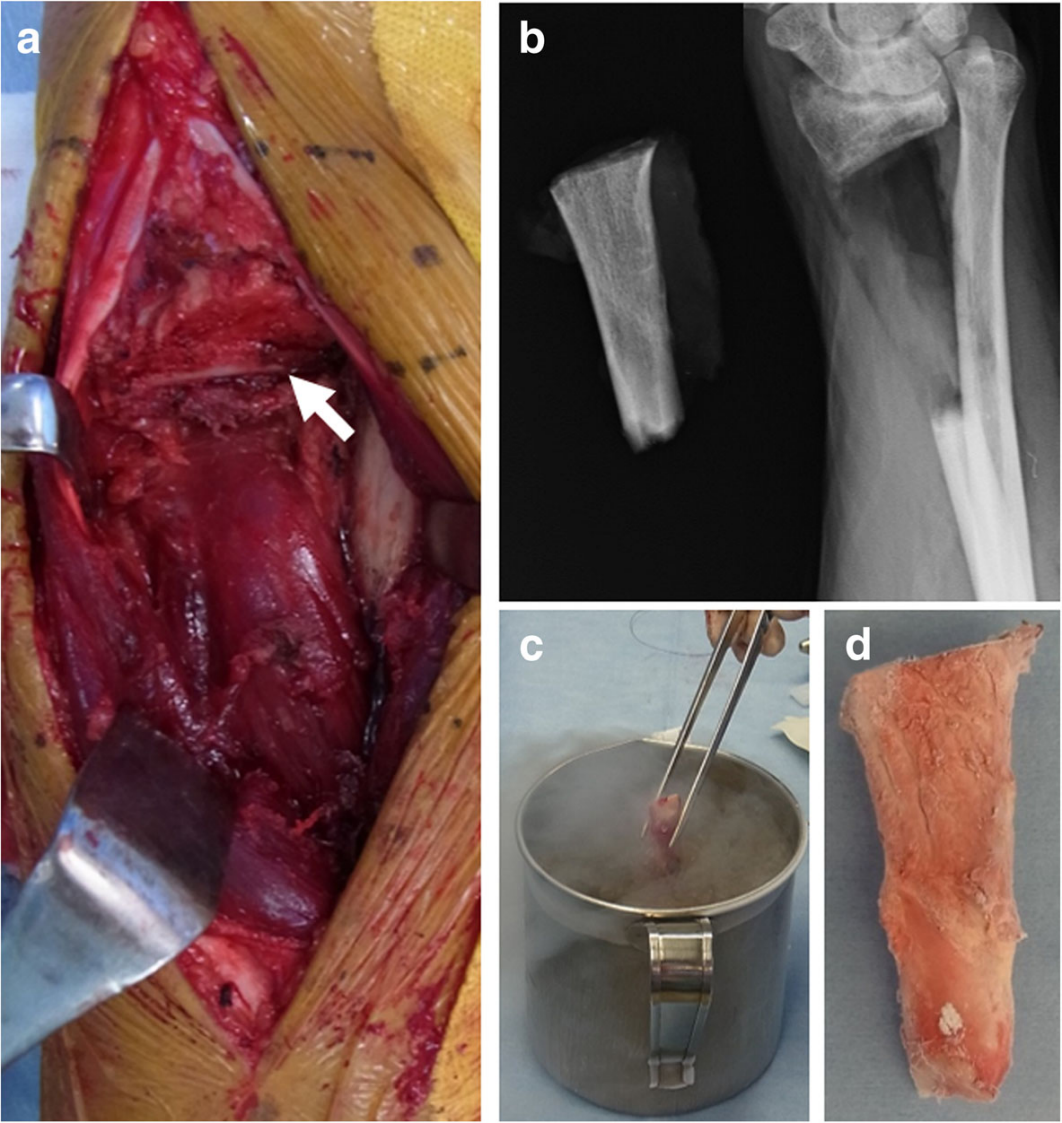
Intraoperative photos (**a**) The dorsal approach at the flexible side of the radiocarpal joint. The tumor bearing bone was resected with a biopsy tract, pronator quadratus muscle, and intraosseous membrane. The main extensor and flexor tendons were preserved. **b** Intraoperative radiograph of the tumor bearing bone resected along the planned osteotomy line. **c** The tumor bearing bone was frozen in liquid nitrogen. **d** After freezing

**Fig. 4:**
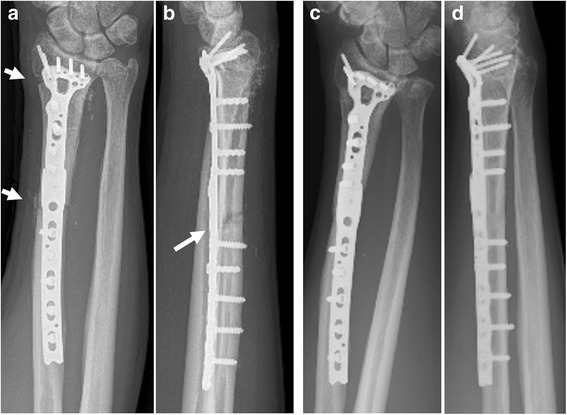
**a**, **b** Postoperative radiographs. Arrows indicate the osteotomy line. **c**, **d** Radiographs of the final follow-up. The bone was completely united and the osteotomy line was obscured

**Fig. 5:**
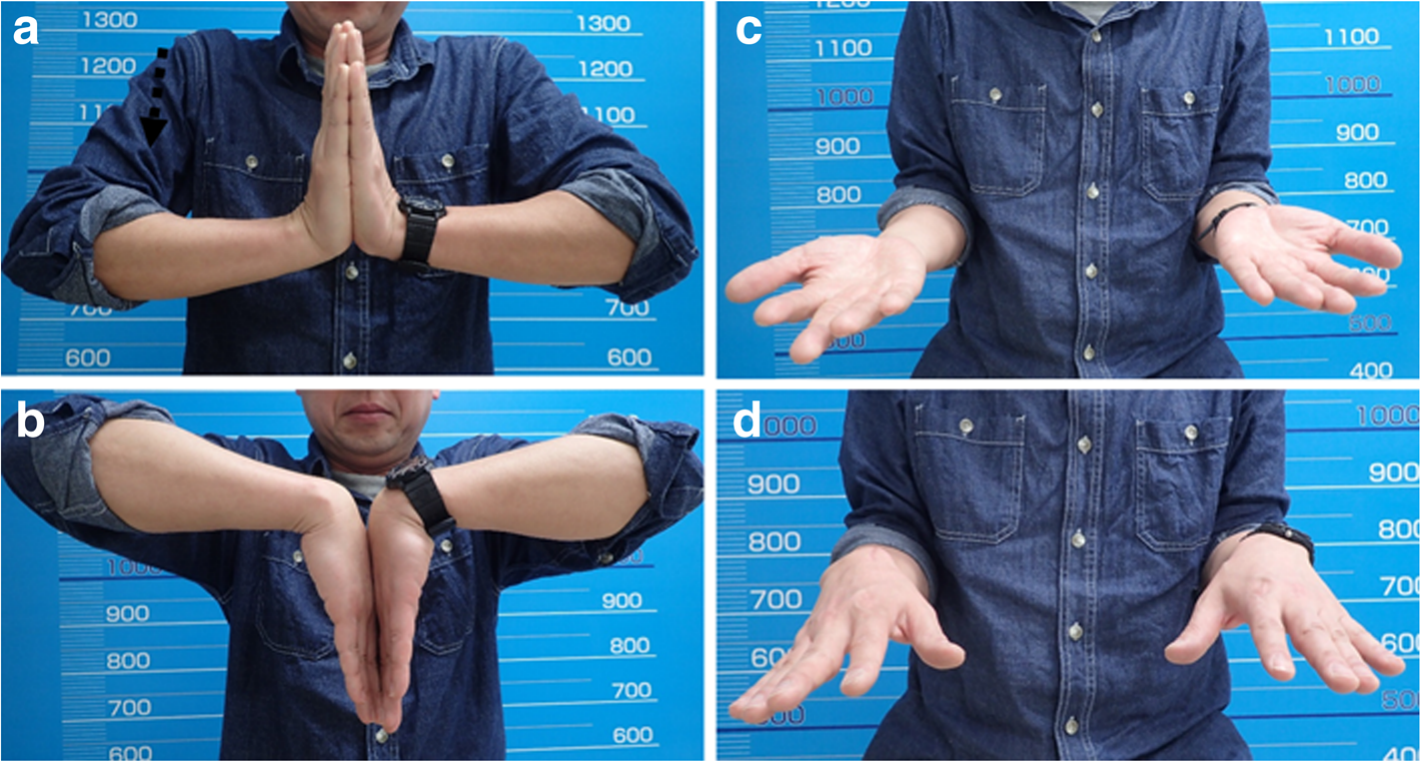
Range of motion of the wrist and forearm at the final follow-up. **a** Dorsiflexion of the right wrist was 85°; (**b**) palmar flexion was 80°; (**c**) pronation of the right forearm was 90°; and (**d**) supination was 90°

A 46-year-old Japanese male patient was referred to our hospital with pain and swelling of the right wrist that had persisted for approximately 3 months. A plain radiograph (Fig. 1a), though hard to discern, and a computed tomography (CT) scan (Fig. 1b, c) of the forearm revealed a periosteal reaction at the dorsal site of the right distal radius. Gadolinium contrast magnetic resonance imaging (MRI) revealed a large soft tissue extension between the radius and the ulna, which was strongly enhanced and continued to an intramedullary enhanced lesion, suggesting that the high-grade malignant tumor arising from intramedullary bone extended widely to the soft tissue. There was no clear bone infiltration into the ulna (Fig. 1d, e). No distant metastasis was detected. The pathological diagnosis from the open biopsy was conventional osteosarcoma (Fig. 1f), and six courses of neoadjuvant chemotherapy with intravenous cisplatin (120 mg/m2) and doxorubicin (30 mg/m2/day × 2 days) were administered according to our chemotherapy regimen [6]. As chemotherapy progressed, the swelling and restricted range of motion (ROM) of the forearm was reduced, with the difference between the left and right wrists disappearing completely after six courses. Preoperatively, the extraosseous lesion appeared greatly shrunken, represented by only a slight signal contrast within the intraosseous membrane. This was confirmed by CT (Fig. 2a) and MRI (Fig. 2b, c), and the response to chemotherapy was considered complete. Wide tumor excision and reconstruction with a tumor bearing frozen autograft was performed. Briefly, a longitudinal dorsal incision was performed and an osteotomy of the distal part of the radius was made 1 cm proximal from the epiphysis to preserve the joint with securing the bony margin according to the preoperative planning using MRI gadolinium enhancement analyses (Fig. 3a). The osteotomy of the proximal part of the radius was made 2 cm proximal from the tumor on the basis of the preoperative MRI and an intercalary resection of the tumor-bearing bone was performed (Fig. 3b). The soft tissue, including the complete pronator quadratus muscle, intraosseous membrane, and periosteum of the ulna where the soft-tissue extension of the tumor originally existed before chemotherapy, was peeled from the ulna and excised with the tumor bearing bone. After stripping the soft tissue, the tumor bearing bone was frozen in liquid nitrogen for 20 min (Fig. 3c, d), thawed at room temperature for 15 min, rinsed in distilled water for 15 min, and then returned to its original site. The frozen autograft was reconstructed with a long volar plate approached from a dorsal incision (Acu-Loc 2 extension plate, Acumed, LLC., OR., USA), and a small volar incision was made in order to insert the distal screws (Fig. 4a, b). Postoperative partial bone union was detected at 3 months and complete bone union was detected at 9 months, both of which were confirmed by plural direction radiography (Fig. 4c, d). Following the surgery, ROM training of both the wrist and fingers commenced immediately and the patient achieved normal ROM with active dorsiflexion of the affected wrist to 85° (Fig. 5a), palmar flexion to 80° (Fig. 5b), and both pronation and supination of the affected forearm to 90° within 9 months after the surgery (Fig. 5c, d). Postoperative functional results were as follows: 100% in the Musculoskeletal Tumor Society (MSTS) score, 12.5 in the Disabilities of the Arm, Shoulder and Hand (DASH) score and 93.5 in the Toronto Extremity Salvage Score (TESS). The Short Form-36 (SF-36) scores accounted for 39.5 of the Physical component summary, 66.6 of the Mental component summary, and 33.4 of the Role/Social component summary at the final postoperative follow-up of 41 months. The patient was discharged after 3 courses of adjuvant chemotherapy. These were administered as neoadjuvant chemotherapy, as the histological evaluation of surgical specimens diagnosed the patient as a good responder (classified as grade III/IV in the Rosen and Huvos evaluation system). At the final follow-up, the patient was found to be disease free.

## Discussion and conclusions

Although the distal radius is an extremely uncommon skeletal site for osteosarcoma, it is not a rare site for benign bone tumors such as giant cell tumors of bone [1]. Even patients with distal radius giant-cell tumors have experienced local recurrence, with subsequent surgeries required following treatment failures. Wang et al. reported that among 27 patients receiving wrist arthrodesis for Campanacci Grade III giant cell tumors of distal radius, 11 patients required additional surgical procedures (41%) (8 for complications (30%) and 3 for local recurrences (11%)) [7]. This finding underscores the difficultly in treating osteosarcomas, very aggressive tumors that require wider margins to treat than giant cell tumors in this site. Many reconstructive procedures for massive defects following the *en bloc* excision of the tumor in the distal radius, including arthrodesis using corticocancellous graft (e.g., iliac crest graft [7], free fibular graft [8, 9]), or ulnar translocation [10]), radial carpal arthroplasty using free fibular head transfer [11, 12], osteoarticular allografts [13], and prosthesis [14, 15] have been reported.

Arthrodesis provides stability at the expense of wrist motion, which may cause impairment in daily life activities. However, if the carpal bones can be preserved, partial arthrodesis (radio-scapho-lunate arthrodesis) is preferred to reduce restrictions in wrist motion to a greater extent than in total arthrodesis [8]. For osteochondral defects, ulnar translocations are less invasive than corticocancellaous grafts in terms of the donor site. However, stress fracture is a potential problem in these grafts, and approximately half of the patients report stress fractures [9].

Several wrist arthroplasty techniques that preserve wrist motion have been reported [11–15]. Wrist prosthesis has been reported and found to result in satisfactory postoperative function [14, 15]. However, limited availability, infection, aseptic loosening, and dorsal subluxation of the ulnar head are the major problems in custom prostheses [15]. Fibular head transfer, along with the shaft, is an attractive method for the replacement of the radiocarpal joint, but the procedure is technically demanding and complications include progressive degenerative changes, bony collapse due to poor vascularity of the fibular head, and volar subluxation resulting from incongruity between the fibular head and the proximal carpal row [12]. These complications, in addition to limited availability, are also problematic in arthroplasty with osteoarticular allografts [13]. Overall, wrist arthroplasty may provide better wrist ROM, at least in the short-term. However, this may come at the expense of wrist stability when compared to wrist arthrodesis. Zhu et al. compared the functional outcomes of 14 distal radius giant cell tumors treated by partial wrist arthrodesis using fibular grafts or arthroplasty using fibular head grafts and found that there were significant differences in flexion-extension; the average wrist ROM of the 7 patients that underwent partial arthrodesis were 55.9° of total flexion-extension and 127.6° of total pronation-supination, whereas the average wrist ROM of the 7 patients that underwent wrist arthroplasty were 71.6° of total flexion-extension and 140° of total pronation-supination [16].

To maintain wrist function and to avoid complications associated with wrist arthrodesis or arthroplasty, preserving the wrist joint by maintaining the epiphysis of the radius is an ideal method in select patients. There is only one report from Yu et al. that mentions wrist joint preserving surgery in osteosarcoma [17]. In this study, the affected limb was reconstructed with a free fibular shaft after *en bloc* intercalary resection of the tumor bone. In a distal radius osteosarcoma case with a short follow-up period, good postoperative function (active dorsiflexion of the affected wrist was to 90° and palmar flexion was to 45°) was found. The author determined that preserving the joint surface could maintain wrist stability and positively affect wrist function [17]. The present case is the second report that involves wrist joint preservation in distal radial osteosarcoma, and the first case to use a recycled bone technique for reconstruction.

The advantages of autograft recycling include perfect fitness to the original site, good availability, lack of the requirement for a bone bank, the capacity for biological reconstruction, lack of disease transmission, reduced immunological response, soft tissue and ligament attachment capabilities, and the availability of a massive bone stock [18]. In 1999, we initially developed our tumor-bearing frozen autograft technique, and have since reported its usefulness [18, 19]. The advantages of frozen autografts include simplicity and the possibility of preserving proteins, including bone morphogenetic proteins (BMPs), which lead to an increased rate of osteoinduction and osteoconduction [20]. In the present study, the frozen bone was united and well preserved at the final follow-up, and the patient was able to achieve normal wrist flexion and extension (dorsiflexion of the affected wrist was to 85° and palmar flexion was to 80°). Furthermore, the patient was able to achieve normal forearm rotation (both pronation and supination to 90°).

Pronation and supination of the forearm play a main role in turning the hand toward an object and are the movements frequently used in daily life [21]. These forearm rotation movements require the anatomical alignment of both the radius and ulna in addition to intact wrist and elbow joints [21, 22]. The misalignment of the radius may lead to radioulnar impingement, increased tension in the interosseous membrane, or contracture of the soft tissues, which have been reported as the main causes for loss of forearm rotation [22, 23]. Using fibular grafting for reconstruction following *en bloc* excision of tumor bone may cause axial malalignment in the forearm, which causes ulnar impingement and worsens forearm rotation [9]. In addition to axial malalignment, shortening of the radius can also induce a severe loss in forearm rotation. Bronstein et al. reported in their cadaveric study that a radial shortening of 10 mm reduced forearm pronation by 47% and supination by 29% [23]. Because the function of the forearm is strongly dependent on the osseous anatomical alignment of the radius, recycled bone, including frozen autografts, can be perfectly matched to the original site and can reproduce the proper anatomical alignment. Therefore, they are more favorable than other reconstructive materials such as fibular allografts or prostheses for reconstruction in forearm tumor surgery. In the present case, we were able to reconstruct the anatomical osseous alignment of the radius by frozen autograft with preservation of the intact ulna, thus enabling the patient to achieve normal forearm rotation.

From the present case, we demonstrated that joint preservation surgery with reconstruction using frozen autografts showed satisfactory results in terms of the function of the affected limb in a case of forearm tumor. However, this technique must be applied to select patients, i.e., those with metaphyseal osteosarcoma with an articular surface, a subchondral bone, a collateral metaphyseal cortex preserved after adequate excision of the tumor, and joints in which internal fixation with plates and screws is possible. In addition, it is important that the patient achieves a good response to neoadjuvant chemotherapy to secure the negative margin. Decke et al., in their series of 39 osteosarcomas of the hand and forearm, reported that the mean overall survival rate in patients with wide or radical tumor resection was higher (88.0%) than in patients in with narrow margins of resection (75.0%) [24]. However, achieving wide tumor resection at the distal forearm is challenging because of the small size of the muscle and expandable soft tissue. Therefore, the surgeon should not hesitate to choose other surgical options, including wrist joint sacrificing surgery or amputation, if the neoadjuvant chemotherapy is not effective or a wide margin is not possible [24]. In the present case, the neoadjuvant chemotherapy was very effective and the soft tissue tumor shrunk remarkably, enabling a secured margin for preservation of the wrist joint, and there were neither local nor distant recurrences found during follow-up.

In conclusion, a wrist joint preservation surgery with tumor bearing frozen autograft reconstruction was successful and resulted in satisfactory functioning of the hand, wrist, and forearm at the final follow-up.

## Abbreviations

CT: Computed tomography
DASH score: Disabilities of the Arm, Shoulder and Hand score
MRI: Magnetic resonance imaging
MSTS score: Musculoskeletal Tumour Society score
ROM: Range of motion
SF-36: Short Form-36
TESS: Toronto Extremity Salvage Score
